# Harm reduction must be recognised an essential public health intervention during crises

**DOI:** 10.1186/s12954-021-00573-6

**Published:** 2021-12-09

**Authors:** Robert Csák, Sam Shirley-Beavan, Arielle Edelman McHenry, Colleen Daniels, Naomi Burke-Shyne

**Affiliations:** Harm Reduction International, 61 Mansell Street, London, UK

**Keywords:** Harm reduction, COVID-19, Public health, Crisis

## Abstract

The COVID-19 had a substantial impact on the provision of harm reduction services for people who use drugs globally. These front-line public health interventions serve a population that due to stigma, discrimination and criminalisation, faces barriers to accessing health and social services and are particularly vulnerable to public health crises. Despite this, the pandemic has seen many harm reduction services close, reduce operations or have their funding reduced. Simultaneously, around the world, harm reduction services have been forced to adapt, and in doing so have demonstrated resilience, flexibility and innovation. Governments must recognise the unique abilities of harm reduction services, particularly those led by the community, and identify them as essential health services that must be protected and strengthened in times of crisis.

## Main text

Harm reduction services for people who use drugs are front-line public health interventions. They serve a population that, due to stigma, discrimination and criminalisation, faces barriers to accessing health and social services and are particularly vulnerable to public health crises. Despite this, across the world states have failed to protect harm reduction services from the impact of the COVID-19 pandemic. Many have been forced to close or seen reductions in their funding. This is counterproductive for public health outcomes. Harm reduction must be recognised as an essential health service that must be protected and strengthened in times of crisis.

People who use drugs must be prioritised in the response to and recovery from COVID-19 because of the unique vulnerabilities many face. People with a long history of opioid or stimulant use are more likely to have a compromised immune system, and people who inject drugs can have underlying medical conditions that make them more vulnerable to certain infectious diseases [1, 2]. Methods of consumption can also mediate risk: smoking or inhaling drugs particularly increases COVID-related risks, as it is associated with pulmonary and respiratory problems [3]. Furthermore, people who use drugs may be more impacted than the general population by quarantine and physical distancing measures in general. They may need to access in-person only harm reduction services like needle and syringe programmes (NSPs) and heavily controlled opioid agonist treatment (OAT) programmes that require daily attendance, or need to frequently procure drugs, again likely in person, to avoid withdrawal symptoms [4]. In addition, social isolation may be associated with increased overdose deaths by increasing the likelihood that people use drugs alone [2].

Far from being prioritised, the pandemic-related restrictions imposed by governments in many countries have had a drastic negative impact on harm reduction service delivery. Around the world, travel restrictions and physical distancing rules meant that services were forced to close or reduce their operating hours, and many were forced to scale back outreach work. In Sub-Saharan Africa, where take-home OAT is rarely available, OAT services were suspended in some countries during COVID-19 and travel to health facilities was restricted [4]. The pandemic seriously affected service delivery and the coverage of harm reduction services in North America, Oceania and Western Europe too, though the impact was less severe compared to other regions [4, 5]. In the USA, harm reduction service providers had to source their own personal protective equipment to ensure safe working environments. In countries in Asia, Eurasia, Latin America and the Middle East and North Africa, government resources were redeployed from harm reduction—signalling its low priority in the minds of policymakers—to fund other areas of the response to COVID-19 [4].

The lack of protection and support from governments meant harm reduction services were forced to adapt and innovate just to maintain their basic services. For example, they extended their online presence, introduced phone and online consultations to replace some face-to-face meetings or utilized social messaging apps to provide counselling or information and keep in touch with clients [4, 6]. Service providers introduced home delivery of harm reduction equipment in Eurasia and Western Europe, and online shops for injecting equipment were set up in the UK and New Zealand [4].

Where governments did act to support harm reduction service delivery during the pandemic, the results had a positive impact. Out of the 84 countries worldwide where OAT is available, 47 countries adapted regulations to expand the availability of take-home OAT by allowing longer take-home periods (by regulation, protocol or in practice). In 23 countries, they made distribution more accessible by permitting home delivery of OAT medication, offering dosing at community pharmacies or distributing in outreach settings. In nine countries they expanded induction practices [4, 6]. Experiences show that these changes were beneficial with high levels of satisfaction among clients and implementers, and should remain in place permanently for clients who prefer the autonomy and flexibility [7]. Longer take-home periods can reduce workload in OAT clinics and decrease barriers in access for people living in remote areas, people with childcare responsibilities and other populations for whom it is difficult to travel, while positively contributing to clients lives without increasing overdoses or diversion [7]. Furthermore, by reducing the burden on clients’ time (such as reducing visits to clinics), take-home OAT can support clients to prioritise personal, family or work commitments [7].

Harm reduction services, particularly community-led services, also demonstrated their great potential in the response to COVID-19 itself. Strong pre-existing relationships with clients meant that they were able to reach sometimes marginalised groups with COVID-19 prevention and care where other services were failing to connect [4]. Integration of harm reduction outreach with COVID-19 prevention and information programmes was widespread. Harm reduction services around the world have delivered hygiene kits, face masks and sanitizer alongside syringes, anti-retrovirals and OAT medication [4]. Some organisations, such as the Tanzanian Network of People who Use Drugs in Dar es Salaam and the Andrey Rylkov Foundation in Russia, also delivered food to clients in self-isolation [6, 8]. This integration of pandemic response with harm reduction services would be an invaluable contribution to any future public health response. It is a further demonstration of the need to prioritise the continuation of harm reduction services in a public health crisis.

## Conclusions

Harm reduction services are innovative, flexible and quick to adapt. They are able to engage a population other services fail to reach. Governments must recognise this and ensure that they are recognised and protected as what they are: essential public health services.

## Data Availability

Not applicable.

## Abbreviations

NSP: Needle and syringe programme
OAT: Opioid agonist therapy
